# Supplementary material for: Analysis of 10,478 cancer genomes identifies candidate driver genes and opportunities for precision oncology
Source: Nat Genet. 2024 Jun 18;56(9):1868–77. doi: 10.1038/s41588-024-01785-9 (PMC11387197; doi:10.1038/s41588-024-01785-9)
Supplement: Supplementary file 1 — Supplementary Note 1 and Figs. 1–9. [file 41588_2024_1785_MOESM1_ESM.pdf]

nature genetics

Supplementary information

https://doi.org/10.1038/s41588-024-01785-9

Analysis of 10,478 cancer genomes identifies candidate driver genes and opportunities for precision oncology

In the format provided by the authors and unedited

Analysis of 10,478 cancer genomes identifies candidate driver genes and opportunities for precision oncology

Supplementary Note 1 - Supplementary Methods

SUPPLEMENTARY METHODS

Curation of clinical data

Clinical data were obtained from Public Health England's National Cancer Registration and Analysis Service (PHE-NCRAS), NHS Digital (NHSD), Genomic Medicine Centres (GMCs) and histology reports via the Genomics England Research Environment. Sequenced samples were matched to their respective PHE-NCRAS records using the date of tumour sampling and the PHE-NCRAS treatment dates, allowing maximum discrepancies of 28 days. Any conflict between data sources was resolved by manual review. Sequenced tumours were assigned to one of 35 tumour groups based on tissue of origin and histology (**Supplementary Tables 1** and **2**).

NHSD and PHE-NCRAS data were used to identify participants that had received systemic treatment or sequenced-cancer-associated radiotherapy before sampling. Records detailing systemic treatments received were obtained from the NHSD admitted patient care and outpatient tables, and the PHE-NCRAS AV Treatment and Systemic Anti-Cancer Therapy (SACT) tables. Records detailing radiotherapy received were obtained from the PHE-NCRAS AV Treatment and National Radiotherapy Dataset (RTDS) tables.

Study sample selection

Tumour samples were excluded if clinical data were missing or if unresolvable conflicts existed between data sources (**Supplementary Table 1**). 2,251 of the 14,129 (15.9%) samples were excluded because: (1) Sex reported by PHE-NCRAS, NHSD and/or the GMC conflicted with the sex inferred from sequencing data; (2) It was not possible to assign the cancer to one of the 35 tumour groups, either because of missing or conflicting originating tissue or histology data, or because the disease was not represented by one group; (3) Ambiguity as to whether a primary tumour, a metastasis or a recurrence of a primary tumour was sampled; (4) It was not possible to assign a date of sampling due to missing or conflicting data; (5) Patient was <18 years old at time of sampling.

Tumour sample purity and sequencing data quality affect variant calling precision and sensitivity and we therefore applied additional quality control (QC) criteria to the WGS data (**Supplementary Table 1**)[1]. Overall, 267/11,878 (2.2%) of tumour samples were excluded based on the following sequencing data QC criteria: (1) Tumour or matched germline cross-contamination was >1%, as determined by VerifyBamID[2]; (2) Number of SNVs called in a tumour was a low outlier for the associated tumour group (SNV number outliers were defined as tumors with a tumor-group-specific log-transformed SNV number Z-score <-3). To ensure that no individual was represented in the same tumour group, we removed duplicated samples from the same individual, keeping primary tumor samples of highest purity, as estimated by Ccube[3]. A further 505 non-solid tumour samples were excluded as these tumour types were not the subject of the present analysis. After imposing these QC metrics, 10,478 tumour samples were suitable for analysis: 9,693 primary tumours, 634 metastases and 151 primary tumour recurrences from 10,470 individuals. Eight patients were represented in more than one tumour group (**Supplementary Table 1**).

Whole genome sequencing

Paired tumour-normal (T/N) samples were obtained as part of the 100kGP programme[4,5]. Recruitment of patients was through 13 Genomic Medicine Centres (GMCs) and affiliated hospitals (**Supplementary Fig. 1**). All patients provided written informed consent. Tissue collection and preparation, extraction and quantification of DNA was undertaken locally, and DNA transferred to a central national biorepository. Whole genome sequencing of paired T/N DNA was conducted by Illumina. Additional processing, quality checking and data storage was performed by Genomics England.

Sample preparation was conducted using Illumina TruSeq DNA PCR-free library preparation kits. Sequencing was performed using HiSeq X, generating 150bp paired-end reads. Tumour and germline samples were sequenced to average depths of 100x and 30x respectively. Poor sequencing quality outliers were identified using principal component analysis and excluded (based on the following quality metrics: average insert size, AT/CG dropout, unevenness of local coverage, percentage of mapped reads and percentage of chimeric DNA fragments). Sequencing

quality outliers were not included in the 100kGP main programme releases and were therefore not considered herein. Reads were aligned to the *Homo sapiens* GRCh38Decoy assembly using Isaac v03.16.02.19[6]. Paired T/N sequencing data for 14,129 cancer samples were obtained from the 100kGP main program version 11 release.

Somatic variant calling

Somatic single nucleotide variant (SNV) and small insertion and deletion (indel) calling was performed using Strelka (v2.4.7)[7]. Variants were excluded if they failed any of the default Strelka filters or met any of the following criteria: (1) population germline allele frequency ≥1% in the gnomAD or 100kGP cohorts[8]; (2) somatic frequency ≥5% in 100kGP tumour samples; (3) overlapped a simple repeat as defined by Tandem Repeats Finder[9]; (4) the indel was in a region with high levels of sequencing noise (high sequencing noise being defined as ≥10% of base calls in a window extending 50 base pairs to either side of the indel call being excluded by Strelka).

SNVs likely to be an artefact caused by systematic mapping or calling issues were identified by computing the ratio of tumour allele depths at each somatic SNV site and comparing against the ratio of allele depths at the same site in a panel of 7,000 normal germline samples. Allele depth at each site was counted, using bcftools mpileup function (version 1.9), considering only individuals not carrying the corresponding alternate allele. Duplicate reads were removed prior to counting and mapping quality ≥5 and base quality ≥5 thresholds were applied to replicate Strelka filters. SNVs with a Phred quality score ≤50 computed using Fisher's exact test were excluded. Copy number alterations (CNAs) were called using Battenberg following variant allele frequency correction with alleleCount-FixVAF[10,11].

Annotation of mutations

Somatic mutations were annotated to GRCh38 Ensembl v101 using the variant effect predictor (VEP)[12]. The following parameters were used: "vep -i <input_vcf> --assembly GRCh38 –no_stats –cache –offline –symbol –protein -o <output> --vcf –canonical –dir <ref_dir> --hgvs –hgvsg –fasta <GRCh38_fasta> --plugin CADD,<CADD_score_file> --plugin

UTRannotator,<GRCh38_uORF_reference>". The <CADD_score_file> was obtained using CADD v1.6 (https://cadd.gs.washington.edu/; with scores obtained for all SNV and indel mutations using the CADD software (https://github.com/kircherlab/CADD-scripts/), before being utilised by the VEP CADD plugin[13–15]. The plugin "UTRannotator" (https://github.com/ImperialCardioGenetics/UTRannotator) was used to annotate the potential impact of five prime untranslated region (5' UTR) mutations[16].

Identification of candidate driver genes

Protein-coding candidate driver genes were identified using the IntOGen pipeline (https://bitbucket.org/intogen/intogen-plus/src/master/; downloaded February 2021; https://intogen.readthedocs.io/en/latest/)[17].

Pre-processing of mutations - Somatic mutations passing filtering criteria described above were subject to initial sample and mutation pre-processing. In the case of multiple tumours from the same patient, the primary tumour was analysed (in the case of primary/recurrence pair), alternatively the tumour with the highest purity was anlaysed. In each cohort, hypermutated tumours were flagged for exclusion from downstream driver gene identification if containing > 10,000 mutations and having an outlier mutation count (count >1.5* interquartile range (IQR) + upper quartile (UQ)). Mutations found to be present in a Hartwig Panel of Normals were further excluded. Unless otherwise specified, mutations were mapped to canonical protein-coding transcripts from Ensembl v101.

Running driver identification methods - Seven complementary driver gene identification methods were run: (1) dNdSCV[18], for the Skin_Melanoma samples which contain a high proportion of hypermutated tumours, the parameter "max_coding_muts_per_sample = Inf" was used; (2) OncodriveFML[18,19], CADD v1.6 scores were used as a measure of functional impact[151314]; (3) OncodriveCLUSTL[20], for the Skin_Melanoma samples, which contain a high proportion of hypermutated tumours, pentamer signatures were used rather than trinucleotide signatures; (4) cBaSE[21]; (5) MutPanning[22]; (6) HotMaps3D[23]; and (7) smRegions[24], this analysis utilised

information from protein family (pfam) domains, which were mapped to Ensembl v101 canonical transcripts.

Combining driver identification methodologies - The driver combination procedure considered the top-100 ranked genes and their association *P*- and *Q*-values in each of the driver identification methods. Briefly, genes assigned as Tier 1 or Tier 2 somatically mutated genes in the COSMIC Cancer Gene Census (https://cancer.sanger.ac.uk/census; v92 downloaded February 2021) were designated as "CGC" genes and represented a "truth" set of known drivers[25]. Through comparison of the relative enrichment of known drivers in the top ranked gene lists a per-method weighting was obtained. Per-method ranked lists were combined using Schulze's voting method to generate a consensus ranking, with combined *P*-values estimated using a weighted Stouffer *Z*-score method.

Driver candidates were classified into the following tiers: Tier 1 – Candidates where the consensus ranking is higher than the ranking of the first gene with Stouffer Q >0.05 (high confidence drivers); Tier 2 – Candidates not meeting the criteria for Tier 1 but which are CGC genes, and show a combined Stouffer Q_{CGC} <0.25 ("rescue" of known cancer drivers); Tier 3 – Candidates not meeting the criteria for Tier 1 or Tier 2 but which have Stouffer Q <0.05 (lower confidence drivers); Tier 4 – Candidates not meeting criteria for Tier 1 or Tier 2 and Stouffer Q >0.05 (candidates not likely to be drivers).

Post-processing of candidate drivers - Candidate driver genes were filtered on the basis of the following annotations:

1. "AUTOMATIC FAIL" – a candidate driver gene would be excluded from further consideration if annotated by at least one of the following:
    a. "TIER4" – categorised into Tier 4 by the combination procedure
    b. "1_METHOD" – only significant (Q <0.1) in 1/7 methods (non-CGC genes)
    c. "EXPRESSION" – gene has very low or absent expression in the relevant The Cancer Genome Atlas (TCGA) Tumor type

d. "OLFACTORY_RECEPTOR" – gene in list of olfactory receptor genes

e. "KNOWN_ARTIFACT" – gene is in a known list of artifacts or long genes (e.g. TTN)

2. "MANUAL REVIEW" – if a gene is not excluded on the basis of any "AUTOMATIC FAIL" filters, it is retained as a candidate driver

   a. "GERMLINE" – non-Tier 1-CGC gene has 1+ mutations per sample and oe_syn/ms/lof > 1.5 based on GnomAD v2.1 constraint metric estimates (https://gnomad.broadinstitute.org/downloads#v2-constraint)

   b. "SAMPLE_3_MUTS" – non-CGC gene where there are 3+ mutations in 1+ Tumor

   c. "LITERATURE" – non-CGC gene where there are no literature annotations according to CancerMine (http://bionlp.bcgsc.ca/cancermine/; downloaded February 2021)[26].

3. "AUTOMATIC PASS" – is not flagged by any "AUTOMATIC FAIL" or "MANUAL REVIEW" filters

Candidate driver roles were assigned on the basis of the dN/dS ratios for missense (w_{mis}) and nonsense (w_{non}) mutations for the given gene derived from dNdSCV (https://bitbucket.org/intogen/intogen-plus/src/master/core/intogen_core/postprocess/drivers/role.py): a "distance" metric was calculated by $\frac{(w_{mis}-w_{non})}{\sqrt{2}}$; candidate drivers where $distance0.1$ represent those with an excess of missense to nonsense mutations and are assigned as "Oncogenes"; candidate drivers where $distance\ < 0.1$ represent those with an excess of nonsense to missense mutations and are assigned as "Tumor suppressor genes (TSGs)"; otherwise, the role of the candidate driver is unclear and was assigned as "Ambiguous".

In the case of multiple cohorts being run representing subsets of a given tumour type, a "consensus" role was designated comparing between each subtype role ("Oncogene" if 1+ cohort and no other cohorts assigned as "TSG", "TSG" if in 1+ cohort and no other cohorts assigned as "Oncogene", otherwise "ambiguous").

Gene candidates were annotated by their overlap with any IntOGen cohorts from a previous 2020.02.01 pan-cancer analysis (https://www.intogen.org/download?file=IntOGen-Cohorts-20200201.zip) and from the pan-cancer TCGA analysis reported by Bailey et al., 2018[27].

Mutations exhibiting extreme strand bias

SNV mutations that otherwise pass filtering criteria as previously detailed were scrutinised if they showed excessive strand bias (*i.e.* Strelka INFO field "SNVSB=" >10). This highlighted the large number of mutations causing the missense change in *CACNA1E (p.Ile95Leu)* as being likely false-positive calls and therefore *CACNA1E* was excluded as a driver candidate. Similarly, we did not consider *WDR64, VCP, GOLGA6L10, NBPF1, TUBB8, TRIM64B, HLA-DQB2* and *KIR3DL2* as being *bona fide* driver genes (**Supplementary Table 14**).

OncoKB annotation of driver mutations

Nonsynonymous mutations in 684 gene transcripts considered by OncoKB v3.11 were annotated using the OncoKB API (https://www.oncokb.org/)[28]. In the first instance, the HGVSg identifier was used, however in rare instances if this failed a combination of gene symbol, consequence and HGVSp were used to map mutations to OncoKB annotations.

Annotation of oncogenic mutations - Nonsynonymous mutations in candidate driver genes were annotated as "Oncogenic" if either of the following criteria were met: (1) the mutation is annotated by OncoKB as "Oncogenic", "Likely Oncogenic" or "Predicted Oncogenic"; (2) the driver role is "Oncogene", consequence is "missense" and mutation is recurrent (seen in >0.5% Tumors pan-cancer); (3) the driver role is "TSG" or "ambiguous" and either the consequence is protein-truncating ("splice acceptor", "splice donor", "frameshift", "stop lost", "stop gained", "start lost") or "missense" and mutation is recurrent (seen in >0.5% Tumors pan-cancer). For POLE, oncogenic annotations were restricted to missense mutations in the exonuclease domain (i.e. amino acid residues 268-471). Nonsynonymous mutations not meeting these criteria were considered as variants of uncertain significance (VUS).

Lollipop plots of driver gene mutations - Lollipop plots of driver gene mutations were generated using the R package trackViewer (https://github.com/jianhong/trackViewer[29]. Pfam protein domains mapping to the Ensembl v101 canonical transcripts were plotted. The protein position was taken from the first position in the HGVSp annotation, other than for splice donor and acceptor mutations where the codon nearest to the HGVSc transcript position was assigned as the protein position.

Power estimates for driver gene estimation - Power to detect a driver assumes a driver gene has a higher non-silent mutation rate compared to the corresponding background mutation rate[30]. A binomial model was used to theoretically compare a driver gene with a non-driver conditional on the sample size. The mutation rate factor, F_g= 3.9, is defined such that driver genes in the 90th percentile of gene-specific background mutation rates (as calculated by MutsigCV[31]) are included. Letting θ be the exome-wide background mutation rate (mutations per base) of a tumour cohort, the general gene mutation background rate for 90th percentile of genes is $\mu = F_g\theta$. r is the non-silent mutation rate above the background rate. The effective gene length is defined as $L_{EFF} = 3L/4$ assuming the ratio of non-silent to silent mutations is 3 to 1 and $L = 1{,}500$ represents the average gene length. The power from the binomial model considers the hypotheses; H_0: $\mu_{NULL} = F_g\theta$, vs H_1: $\mu_{ALT} = 1 - ((1-\mu_{NULL})^{L_{EFF}} - r)^{(1/L_{EFF})}$, where μ_{ALT} is the non-silent mutation rate of a suspected driver gene. The null hypothesis is rejected at a *P*-value of 5×10^{-6}.

Comparison of candidate driver gene mutation rates in different histologies

To compare the rate of driver somatic mutations in different histologies per cancer tissue, expected mutation rate and uncertainties were estimated by taking a uniform distribution prior with a binomial likelihood function with the number of samples which are mutated (k) vs total samples (n) for the given cancer site. This generates a beta distribution posterior with parameters $\alpha = k + 1$, $\beta = n - k + 1$ such that the expected mutation rate is $(k+1)/(n+2)$. For each histology, *P*-values are evaluated using a binomial test of the mutation rate of the samples with the given histology against the mutation rate of all samples for the given organ.

Assessment of domain specific mutations

We assessed domain specific mutations by considering the cancer drivers where smRegions is a significant bidder (*Q*-value <0.1) and the driver is annotated in multiple cancer types.

Predicting mismatch repair deficiency

Samples with evidence of defective mismatch repair were detected using mSINGS, following the previously described procedure for background model generation[32,33].

Copy number profiling and whole genome doubling Clonal and subclonal somatic copy number alterations (CNAs) were identified using an iterative process incorporating Battenberg v2.2.8[11]. Whole genome duplicated tumours were identified based on the methodology described in Gerstung, et al. (2020)[34]. Further details can be found in our accompanying manuscripts[32,35].

Structural variants calling

We identified structural variants using a graph-based consensus approach including Delly, Lumpy and Manta, and support from CNAs[36–38]. Additional details can be found in our accompanying manuscripts[32,35].

Predicting homologous recombination deficiency

Evidence of homologous recombination deficiency (HRD) was assessed using HRDetect[39]. HRDetect requires CNA data and was therefore run only on 9,207 tumours passing CNA calling. To compute the HRDetect score we determined the following input features: exposures of single base substitution signatures, SBS3 and SBS8, as well as COSMIC rearrangement signatures 3 and 5, the proportion of short deletions at microhomology, and the HRD-LOH index[39,40]. SBS3 and SBS8 contribution estimates were obtained from SigProfiler[41]. We used a probabilistic cutoff of 0.7, which translates to 98.7% sensitivity for predicting BRCA1/BRCA2 deficiency and has a high efficacy when applied to multiple cancer types[39].

Comparison of driver mutation frequencies between Genomics England and MSK

To determine the sensitivity of WGS data from the 100kGP we first compared driver mutation frequencies with those from MSK-IMPACT and MSK-MET, a combined cohort of ~25,000 cancer patients whose tumours have been panel sequenced to identify driver mutations[42,43]. Mutation and sample data were downloaded from https://cbioportal-datahub.s3.amazonaws.com/msk_impact_2017.tar.gz and https://cbioportal-datahub.s3.amazonaws.com/msk_met_2021.tar.gz. Where possible, MSK tumours were matched to 100kGP tumour groupings on the basis of their oncotree code. For CNS tumours, IDH mutation status was used to classify GBM tumours as GBM, IDHwt or GBM, IDmut. Due to lack of 1p/19q co-deletion status, CNS tumours classified as mixed oligoastrocytomas were excluded. To maintain consistency with 100kGP, MSK mutations were lifted over to GRCh38 and annotated by VEP v101 and OncoKB. Samples were aggregated by tumour group and type (metastasis/primary) and the fraction of samples with an oncogenic mutation in a given driver gene were compared.

Assessing the sensitivity of WGS to detect driver mutations

By analysing the distribution of allelic depths in called PASS mutations in the 100kGP cohort we found that the rate of calls falls when <6 reads support the alternate allele (**Supplementary Fig. 13**). We therefore used 6 reads as a minimum coverage threshold to approximate the sensitivity of the WGS samples to somatic mutations. Per-base coverage was extracted from tumour bam files using GATK v4.4.0.0 DepthOfCoverage[44]. A gene panel of 43 representative driver genes was obtained from the NHS Genomic Test Directory for Cancer (2021-22 v5.0 published 31 October 2022). Genomic regions were defined as per driver gene identification (*i.e.* coding sequence (CDS) from the canonical ensembl v101 transcript including essential splice sites). The *TERT* promoter region was defined as GRCh38 chr5:1295019-1295268. Coverage was mapped to gene panel regions using bedops v2.4.26 and bedtools v2.3.0 to obtain per-gene coverage statistics[45,46]. Assuming a heterozygous clonal mutation the expected number of reads is given by $0.5 \times coverage \times purity$. The distribution of coverage across samples for three common pancancer drivers is given in **Supplementary Fig. 14**. The sensitivity is the probability of measuring at least 6 reads given the expected number of reads which we assume is Poisson distributed. We estimated this for all genes and samples shown in **Supplementary Fig. 15 & 16**.

In a realistic worst case, for a read coverage of 75 (i.e. lower 5th percentile of genes in samples) with a tumour purity of 0.2, the sensitivity is 76% however, this rises to 99.98% for a purity of 0.5. We also estimated the fraction of each driver gene with an expected alt read count > 6. Results for *TP53, KRAS* and *PIK3CA* are given in **Supplementary Fig. 17**.

Comparison of actionability between WGS and panel

Actionable driver genes were defined from the OncoKB and COSMIC databases. This list was compared with the NHS Genomic Test Directory for Cancer (2021-22 v5.0 published 31 October 2022) at a mutation-specific level.

Timing candidate driver gene mutations

The relative evolutionary timing of candidate driver mutations was obtained using MutationTimeR (https://github.com/gerstung-lab/MutationTimeR)[34]. MutationTimeR times somatic mutations relative to clonal and subclonal copy number states and calculates the relative timing of copy number gains. Hence, the number of clonal and subclonal copy number states can influence estimates of the timing of somatic mutations across different tumor types.

Preparing MutationTimeR input files

Copy number input for MutationTimeR was prepared from Battenberg segmentation files, with the clonal frequency of each segment taken as the tumour purity. In the case of subclonal calls, the clonal frequency was calculated by multiplying the tumour purity by the clonal fraction.

The clusters input for MutationTimeR was prepared from DPClust cluster estimates. The VAF proportion was calculated by multiplying the estimated cluster CCF by the tumour purity. Superclonal clusters (CCF >1.1) were removed.

VCF input for MutationTimeR was obtained from the small somatic SNV/indel variant VCFs which had been filtered as previously described. For SNVs, alt and ref depths were obtained using FixVAF (https://github.com/danchubb/FixVAF). For indels, ref and alt depths were obtained from

Tier2 Strelka TAR and TIR fields respectively. Only mutations within Battenberg copy-number segments were retained (note: for male XY tumours with only 1 copy of the X chromosome copy number information is restricted to the pseudoautosomal region (PAR) and battenberg was not run on the Y chromosome).

Running MutationTimeR

MutationTimeR was run with 1,000 bootstraps. For tumours previously defined as having undergone whole genome doubling (WGD), the parameter “isWgd” was set to TRUE. Mutations were then classified into estimated simple clonal states (as per Fig.1a of Gerstung *et al.* (2020)[34]):

- “Clonal [early]” – Mutation on ≥2 copies per cell
- “Clonal [late]” – Mutation on 1 copy per cell, no retained allele
- “Clonal [NA]” – Mutation on 1 copy per cell, either on amplified or retained allele
- “Subclonal” – Mutation on <1 copy per cell

Creating a canSAR interactome

The canSAR interactome features interactions where there are: (i) at least two publications with experimental evidence of binary interaction between the two proteins; (ii) 3D protein evidence of a complex; (iii) at least two reports that one protein is a substrate of the other; (iv) at least two publications reporting that one protein is the product of a gene under the direct regulatory control of the other. Each tumour-specific interactome was seeded using cancer driver proteins, retrieving interacting proteins that had supporting experimental evidence. To ensure that additional proteins are likely to function primarily through interaction with proteins in the network, we adopted the following strategy: starting with the input list of proteins we obtained all possible first neighbours. We then computed, for each new protein the proportion of its first neighbours in the original input list. To define proteins likely to function through the network, we calculated the probability of these occurring randomly by permuting the interactome 10,000 times. We corrected empiric *P*-values for multiple testing retaining only proteins having a FDR <

0.05. For each cancer type we minimised the network by retaining only proteins connected to more than one cancer protein, or whose only connection was to a cancer-specific protein.

REFERENCES

1. Saunders, C. T. *et al.* Strelka: accurate somatic small-variant calling from sequenced tumor-normal sample pairs. *Bioinformatics* **28**, 1811–1817 (2012).
2. Jun, G. *et al.* Detecting and estimating contamination of human DNA samples in sequencing and array-based genotype data. *Am. J. Hum. Genet.* **91**, 839–848 (2012).
3. Yuan, K., Macintyre, G., Liu, W., Markowetz, F. & PCAWG-11 working group. Ccube: A fast and robust method for estimating cancer cell fractions. Preprint at https://doi.org/10.1101/484402.
4. Turnbull, C. Introducing whole-genome sequencing into routine cancer care: the Genomics England 100 000 Genomes Project. *Ann. Oncol.* **29**, 784–787 (2018).
5. Turnbull, C. *et al.* The 100 000 Genomes Project: bringing whole genome sequencing to the NHS. *BMJ* k1687 Preprint at https://doi.org/10.1136/bmj.k1687 (2018).
6. Raczy, C. *et al.* Isaac: ultra-fast whole-genome secondary analysis on Illumina sequencing platforms. *Bioinformatics* **29**, 2041–2043 (2013).
7. Kim, S. *et al.* Strelka2: fast and accurate calling of germline and somatic variants. *Nat. Methods* **15**, 591–594 (2018).
8. Karczewski, K. J. *et al.* The mutational constraint spectrum quantified from variation in 141,456 humans. *Nature* **581**, 434–443 (2020).
9. Benson, G. Tandem repeats finder: a program to analyze DNA sequences. *Nucleic Acids Research* vol. 27 573–580 Preprint at https://doi.org/10.1093/nar/27.2.573 (1999).
10. Cornish, A. J. *et al.* Reference bias in the Illumina Isaac aligner. *Bioinformatics* vol. 36

4671–4672 (2020).

11. Nik-Zainal, S. *et al.* Mutational processes molding the genomes of 21 breast cancers. *Cell* **149**, 979–993 (2012).

12. McLaren, W. *et al.* The Ensembl Variant Effect Predictor. *Genome Biol.* **17**, 122 (2016).

13. Rentzsch, P., Witten, D., Cooper, G. M., Shendure, J. & Kircher, M. CADD: predicting the deleteriousness of variants throughout the human genome. *Nucleic Acids Res.* **47**, D886–D894 (2019).

14. Rentzsch, P., Schubach, M., Shendure, J. & Kircher, M. CADD-Splice-improving genome-wide variant effect prediction using deep learning-derived splice scores. *Genome Med.* **13**, 31 (2021).

15. Kircher, M. *et al.* A general framework for estimating the relative pathogenicity of human genetic variants. *Nat. Genet.* **46**, 310–315 (2014).

16. Zhang, X., Wakeling, M., Ware, J. & Whiffin, N. Annotating high-impact 5'untranslated region variants with the UTRannotator. *Bioinformatics* **37**, 1171–1173 (2021).

17. Martínez-Jiménez, F. *et al.* A compendium of mutational cancer driver genes. *Nat. Rev. Cancer* **20**, 555–572 (2020).

18. Martincorena, I. *et al.* Universal Patterns of Selection in Cancer and Somatic Tissues. *Cell* **171**, 1029–1041.e21 (2017).

19. Mularoni, L., Sabarinathan, R., Deu-Pons, J., Gonzalez-Perez, A. & López-Bigas, N. OncodriveFML: a general framework to identify coding and non-coding regions with cancer driver mutations. *Genome Biol.* **17**, 128 (2016).

20. Arnedo-Pac, C., Mularoni, L., Muiños, F., Gonzalez-Perez, A. & Lopez-Bigas, N.

OncodriveCLUSTL: a sequence-based clustering method to identify cancer drivers. *Bioinformatics* **35**, 4788–4790 (2019).

21. Weghorn, D. & Sunyaev, S. Bayesian inference of negative and positive selection in human cancers. *Nat. Genet.* **49**, 1785–1788 (2017).

22. Dietlein, F. *et al.* Identification of cancer driver genes based on nucleotide context. *Nat. Genet.* **52**, 208–218 (2020).

23. Tokheim, C. *et al.* Exome-Scale Discovery of Hotspot Mutation Regions in Human Cancer Using 3D Protein Structure. *Cancer Res.* **76**, 3719–3731 (2016).

24. Porta-Pardo, E. & Godzik, A. e-Driver: a novel method to identify protein regions driving cancer. *Bioinformatics* **30**, 3109–3114 (2014).

25. Sondka, Z. *et al.* The COSMIC Cancer Gene Census: describing genetic dysfunction across all human cancers. *Nat. Rev. Cancer* **18**, 696–705 (2018).

26. Lever, J., Zhao, E. Y., Grewal, J., Jones, M. R. & Jones, S. J. M. CancerMine: a literature-mined resource for drivers, oncogenes and tumor suppressors in cancer. *Nat. Methods* **16**, 505–507 (2019).

27. Bailey, M. H. *et al.* Comprehensive Characterization of Cancer Driver Genes and Mutations. *Cell* **173**, 371–385.e18 (2018).

28. Chakravarty, D. *et al.* OncoKB: A Precision Oncology Knowledge Base. *JCO Precis Oncol* **2017**, (2017).

29. Ou, J. & Zhu, L. J. trackViewer: a Bioconductor package for interactive and integrative visualization of multi-omics data. *Nat. Methods* **16**, 453–454 (2019).

30. Tokheim, C. J., Papadopoulos, N., Kinzler, K. W., Vogelstein, B. & Karchin, R. Evaluating the

evaluation of cancer driver genes. *Proc. Natl. Acad. Sci. U. S. A.* **113**, 14330–14335 (2016).

31. Lawrence, M. S. *et al.* Discovery and saturation analysis of cancer genes across 21 tumour types. *Nature* **505**, 495–501 (2014).
32. Cornish, A. J. *et al.* Whole genome sequencing of 2,023 colorectal cancers reveals mutational landscapes, new driver genes and immune interactions. *bioRxiv* 2022.11.16.515599 (2022) doi:10.1101/2022.11.16.515599.
33. Salipante, S. J., Scroggins, S. M., Hampel, H. L., Turner, E. H. & Pritchard, C. C. Microsatellite instability detection by next generation sequencing. *Clin. Chem.* **60**, 1192–1199 (2014).
34. Gerstung, M. *et al.* The evolutionary history of 2,658 cancers. *Nature* **578**, 122–128 (2020).
35. Everall, A. *et al.* Comprehensive repertoire of the chromosomal alteration and mutational signatures across 16 cancer types from 10,983 cancer patients. *bioRxiv* (2023) doi:10.1101/2023.06.07.23290970.
36. Rausch, T. *et al.* DELLY: structural variant discovery by integrated paired-end and split-read analysis. *Bioinformatics* **28**, i333–i339 (2012).
37. Layer, R. M., Chiang, C., Quinlan, A. R. & Hall, I. M. LUMPY: a probabilistic framework for structural variant discovery. *Genome Biol.* **15**, R84 (2014).
38. Chen, X. *et al.* Manta: rapid detection of structural variants and indels for germline and cancer sequencing applications. *Bioinformatics* **32**, 1220–1222 (2016).
39. Davies, H. *et al.* HRDetect is a predictor of BRCA1 and BRCA2 deficiency based on mutational signatures. *Nat. Med.* **23**, 517–525 (2017).
40. Nik-Zainal, S. *et al.* Landscape of somatic mutations in 560 breast cancer whole-genome sequences. *Nature* **534**, 47–54 (2016).

41. Ashiqul Islam, S. M. *et al. Uncovering Novel Mutational Signatures by de Novo Extraction with SigProfilerExtractor*. (2022).

42. Zehir, A. *et al.* Mutational landscape of metastatic cancer revealed from prospective clinical sequencing of 10,000 patients. *Nat. Med.* **23**, 703–713 (2017).

43. Nguyen, B. *et al.* Genomic characterization of metastatic patterns from prospective clinical sequencing of 25,000 patients. *Cell* **185**, 563–575.e11 (2022).

44. McKenna, A. *et al.* The Genome Analysis Toolkit: a MapReduce framework for analyzing next-generation DNA sequencing data. *Genome Res.* **20**, 1297–1303 (2010).

45. Neph, S. *et al.* BEDOPS: high-performance genomic feature operations. *Bioinformatics* **28**, 1919–1920 (2012).

46. Quinlan, A. R. & Hall, I. M. BEDTools: a flexible suite of utilities for comparing genomic features. *Bioinformatics* **26**, 841–842 (2010).

Analysis of 10,478 cancer genomes identifies candidate driver genes and opportunities for precision oncology

Supplementary Figures

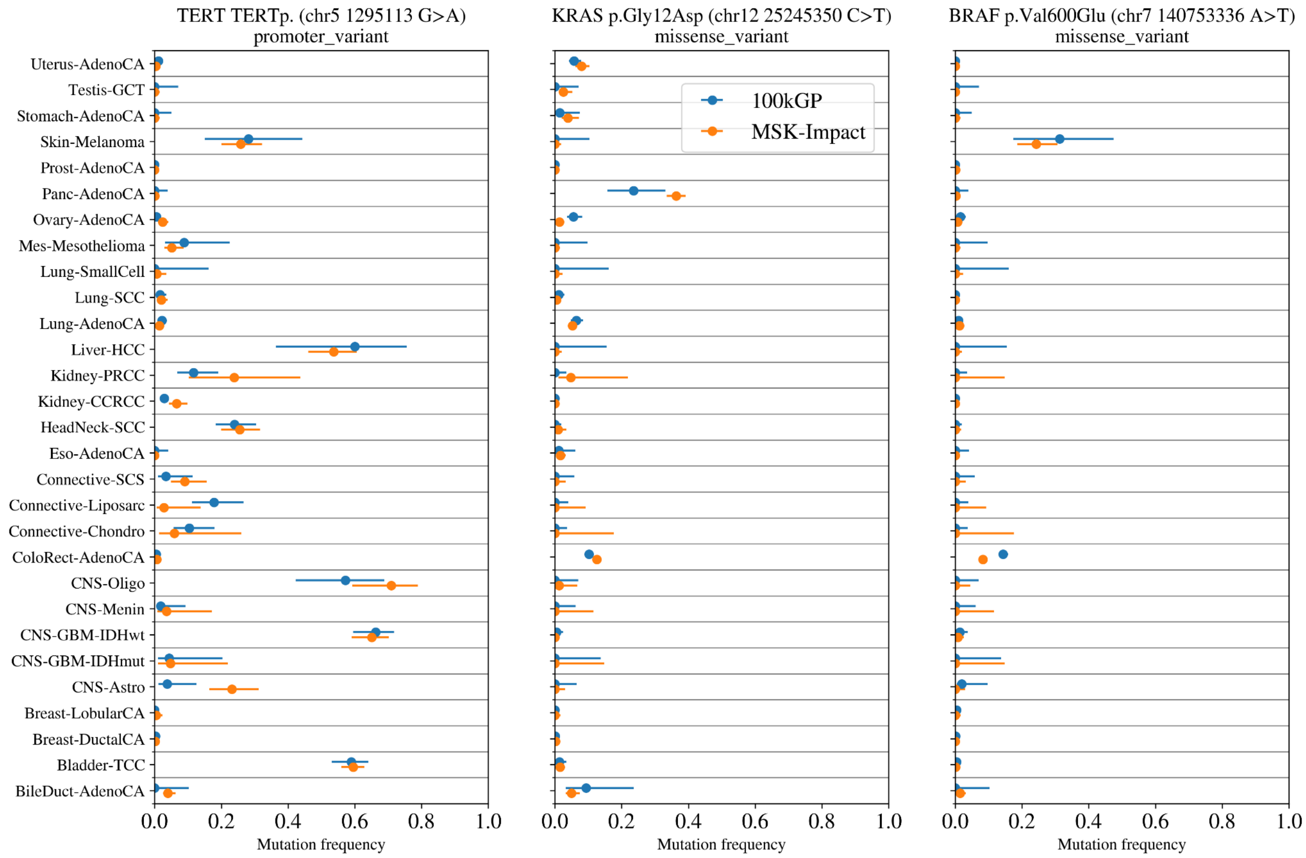

Supplementary Figure 1. The rate of hotspot mutations in the 100kGP sample compared with panel sequencing in MSK IMPACT study. The fraction of the samples with the hotspot mutation with 95% confidence intervals (binomial distribution with uniform prior). Data from 100kGP cohort consists of 10,478 tumours (34 bile duct, 305 bladder, 2,306 breast, 2,324 colorectal, 440 central nervous system, 91 esophageal, 201 head and neck, 1,045 renal cell, 24 liver, 1,110 lung, 35 mesothelioma , 607 soft-tissue, 454 ovarian, 94 pancreas, 366 prostate, 270 melanoma, 72 gastric, 51 testicular, 649 uterus).

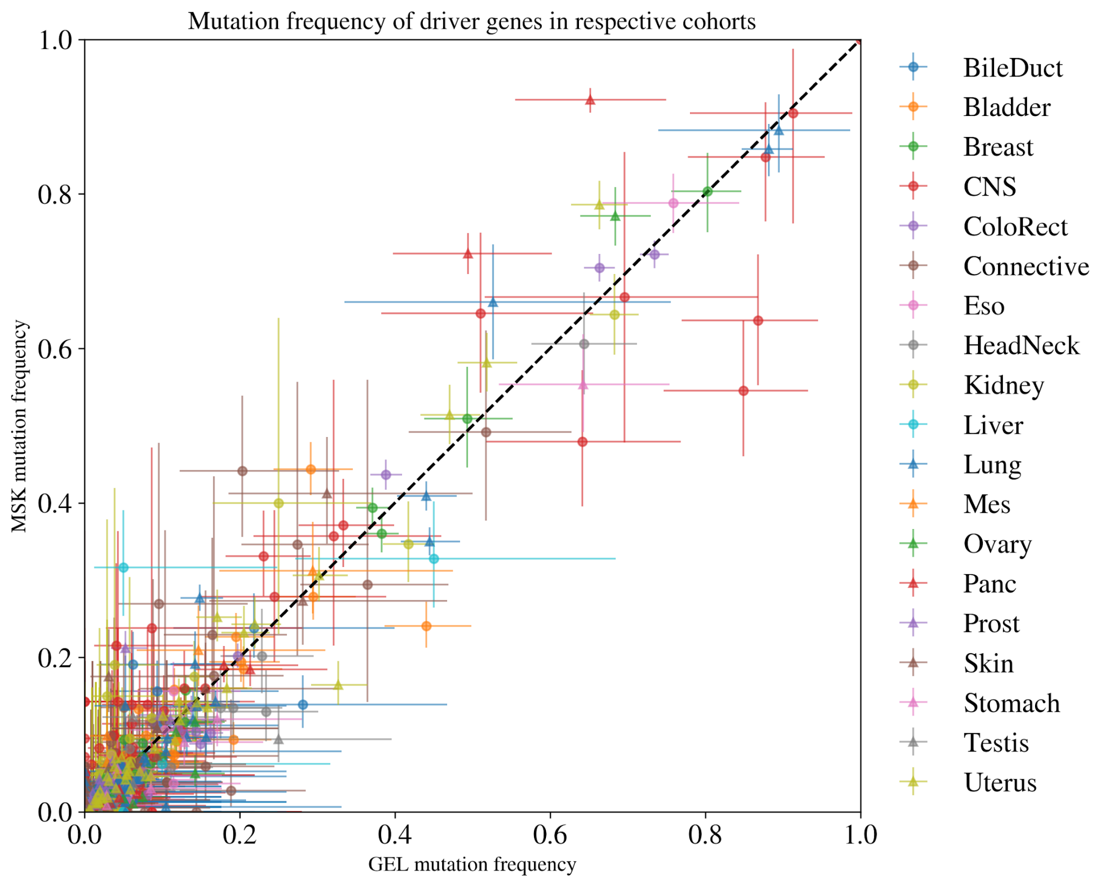

Supplementary Figure 2. The rate of mutations in candidate driver genes in the100kGP cohort compared to the MSK cohort. The fraction of the samples with a hotspot mutation in the tumour group (95% confidence intervals for a binomial distribution with uniform prior). Each point corresponds to one of the 770 driver genes in tumour groups where the colour and point shape corresponds to the organ of the tumour group. Data from 100kGP cohort consists of 10,478 tumours (34 bile duct, 305 bladder, 2,306 breast, 2,324 colorectal, 440 central nervous system, 91 esophageal, 201 head and neck, 1,045 renal cell, 24 liver, 1,110 lung, 35 mesothelioma , 607 soft-tissue, 454 ovarian, 94 pancreas, 366 prostate, 270 melanoma, 72 gastric, 51 testicular, 649 uterus).

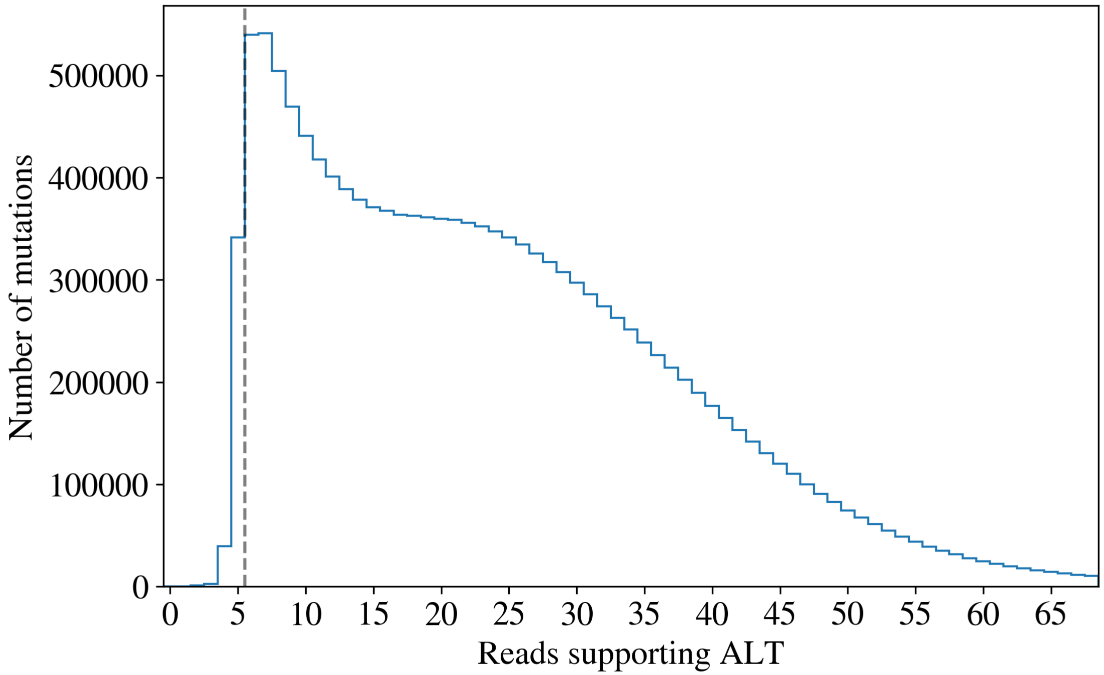

Supplementary Figure 3. Distribution of read counts supporting the alternate allele across all PASS mutations in all tumour samples. The steep dropoff in called mutations with fewer than 6 reads supporting the alternate allele suggests that a threshold of 6 is a reasonable proxy for selection criteria of Strelka variant calls.

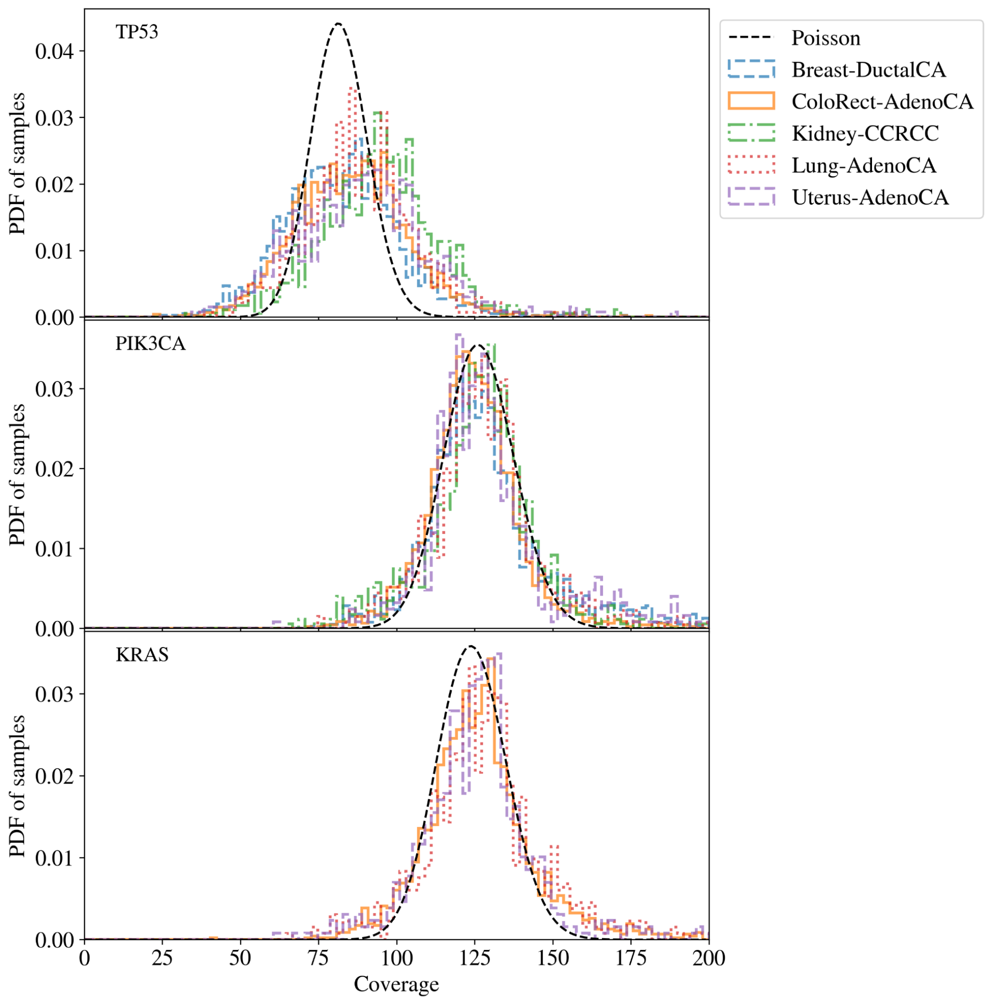

Supplementary Figure 4. The distribution average read coverage for each sample in the given tumour group for the given gene. Most genes such as PIK3CA and KRAS have greater than 100x coverage in the majority of samples and spread consistent with random Poisson noise. TP53 has significantly lower and more varied coverage between samples.

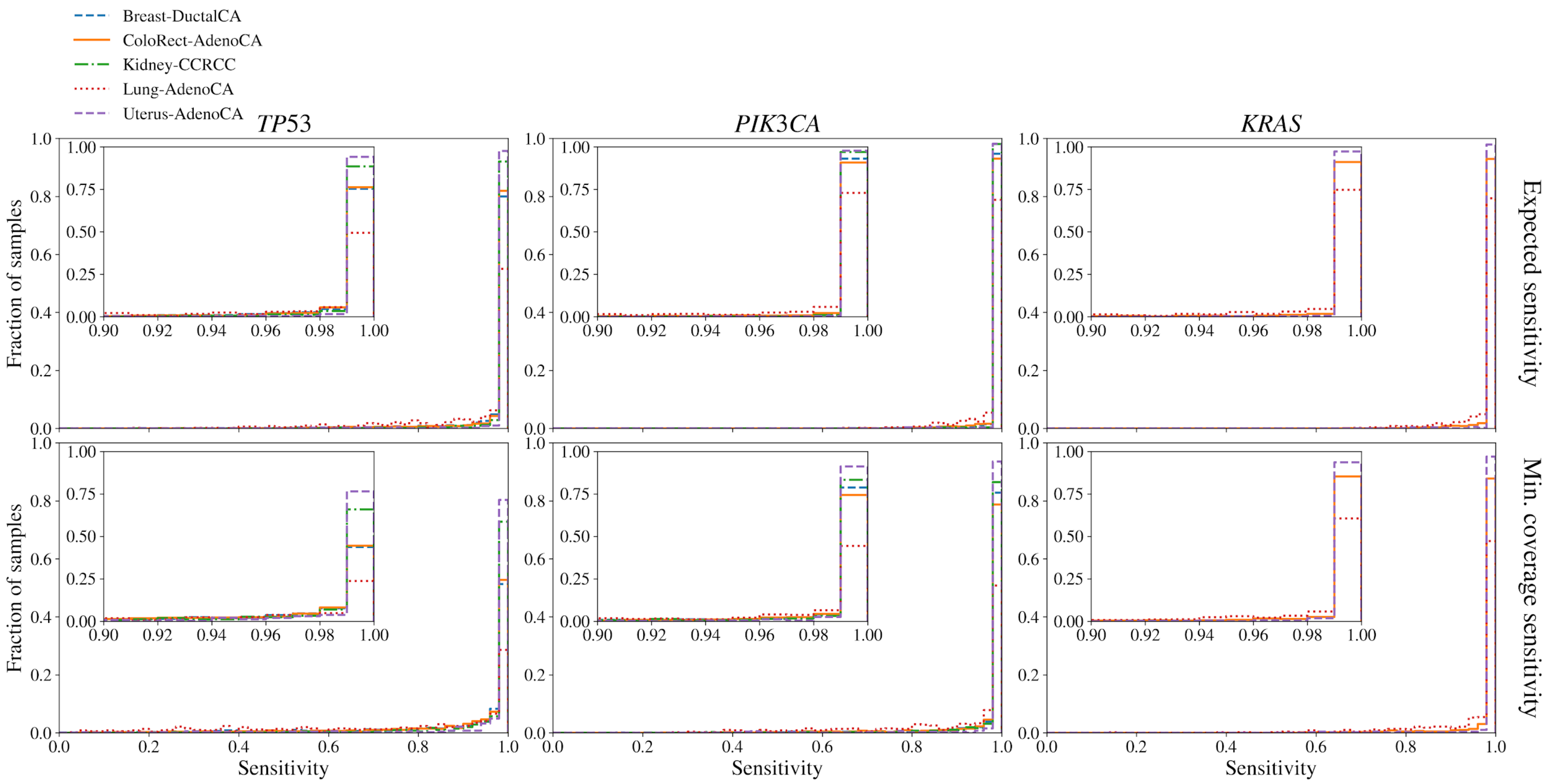

Supplementary Figure 5. Distribution of mean sensitivity (top) and minimum sensitivity (bottom) across samples in the given tumour group for the given gene. The mean sensitivity is estimated as the Poisson probability that there will be at least 6 alternate allele reads given the purity and mean gene coverage for the sample while the minimum sensitivity is given the minimum coverage across the gene. Across 88% of genes and samples have greater than 99% mean sensitivity.

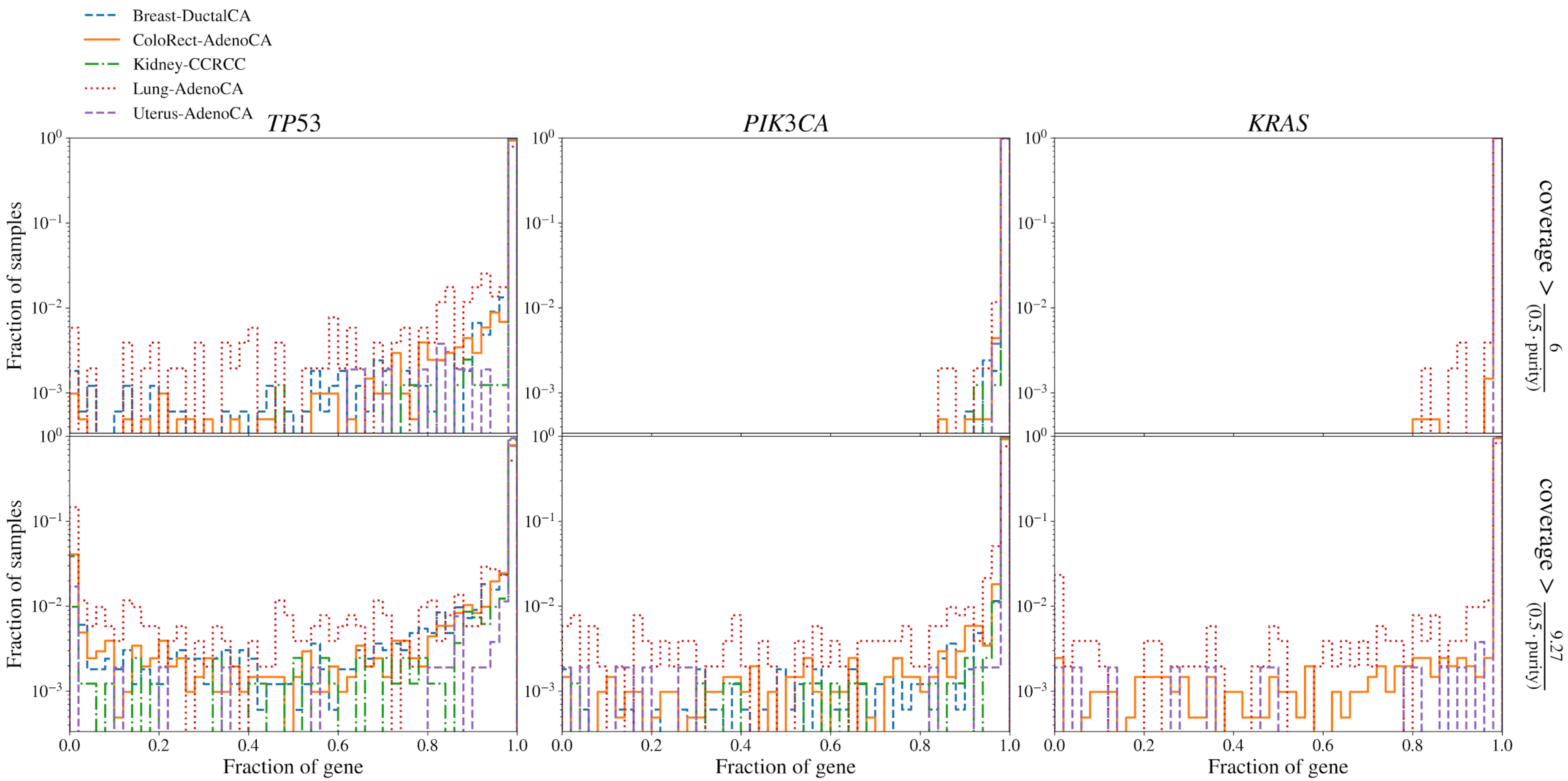

Supplementary Figure 6. Fraction of the gene where the coverage is high enough that the sensitivity would be at least 50% (top) and 90% (bottom). The expected read count supporting alternate alleles is given by $0.5 \times coverage \times purity$. 6 alternate reads would give approximately a 50% chance of detecting a variant and 9.27 reads corresponds to approximately 90%. In 90% of all genes and samples, over 98% of the gene has sufficient coverage and purity to have an expected alternate read count greater than 6.

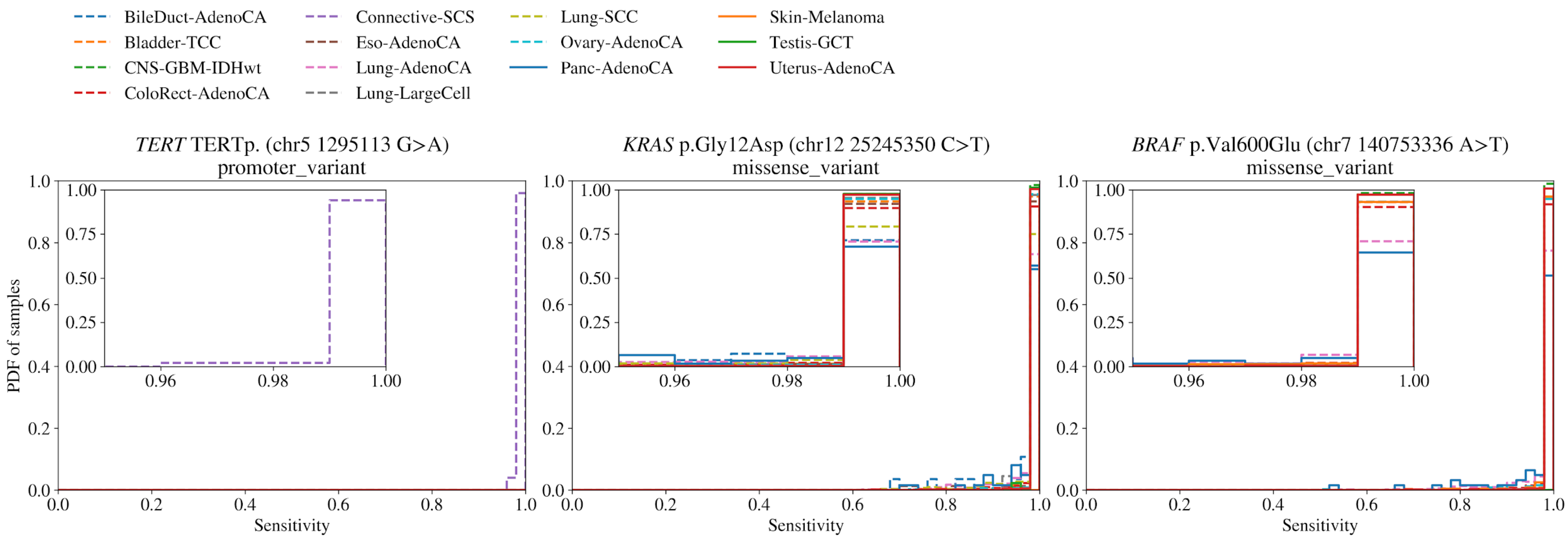

Supplementary Figure 7. The distribution of expected sensitivity to hotspot mutations across samples in the given tumour group. The sensitivity is estimated based on the Poisson probability of there being at least 6 alternate allele reads given the coverage and sample purity. 88% of hotspots in samples have greater than 99% sensitivity.

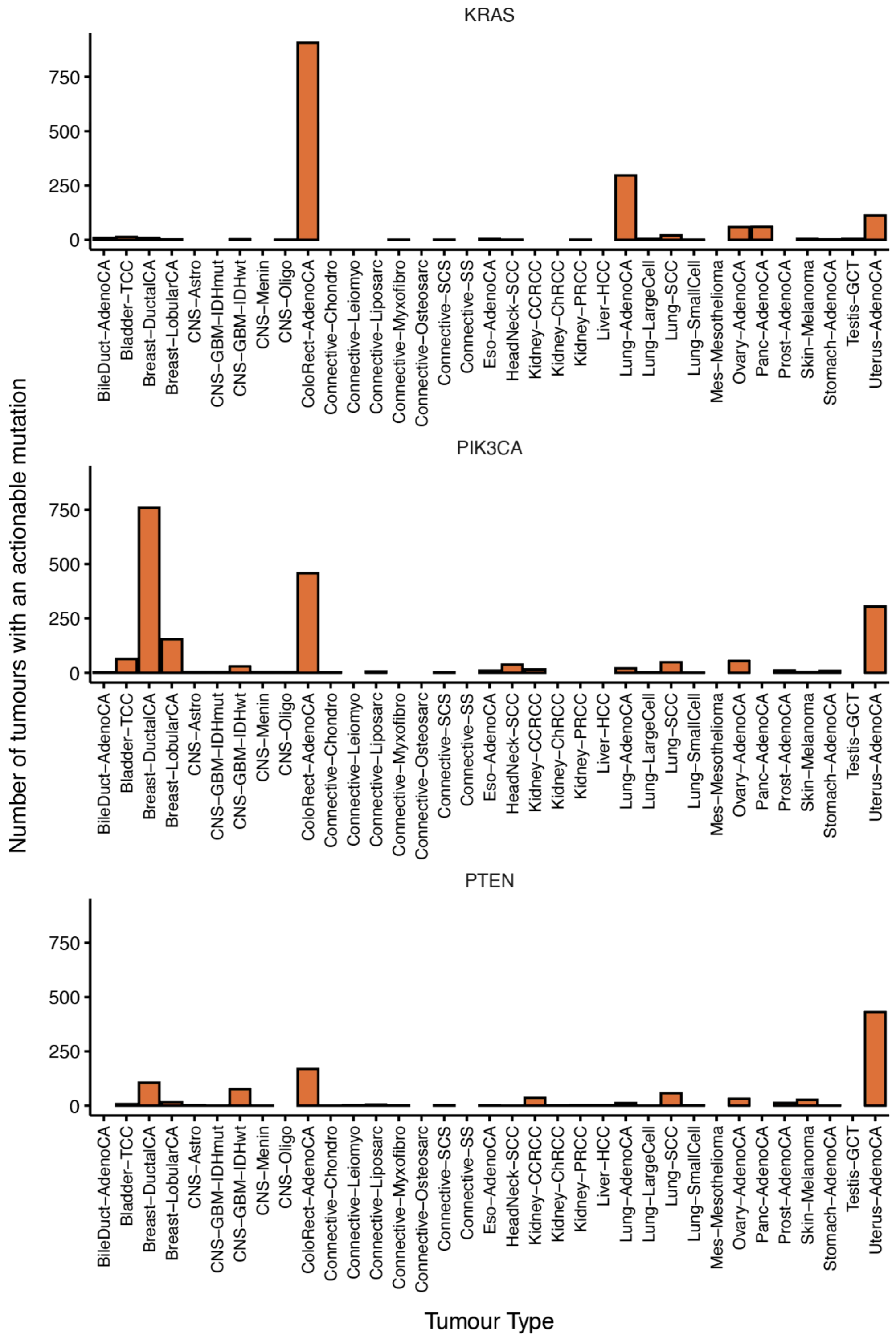

Supplementary Figure 8. Number of samples in tumour group with actionable mutations in *KRAS*, *PIK3CA* and *PTEN*. Actionability defined by OncoKB and includes FDA approved drug, standard of care or clinical evidence in the cancer type, standard of care in a different cancer type or supported by compelling biological evidence.

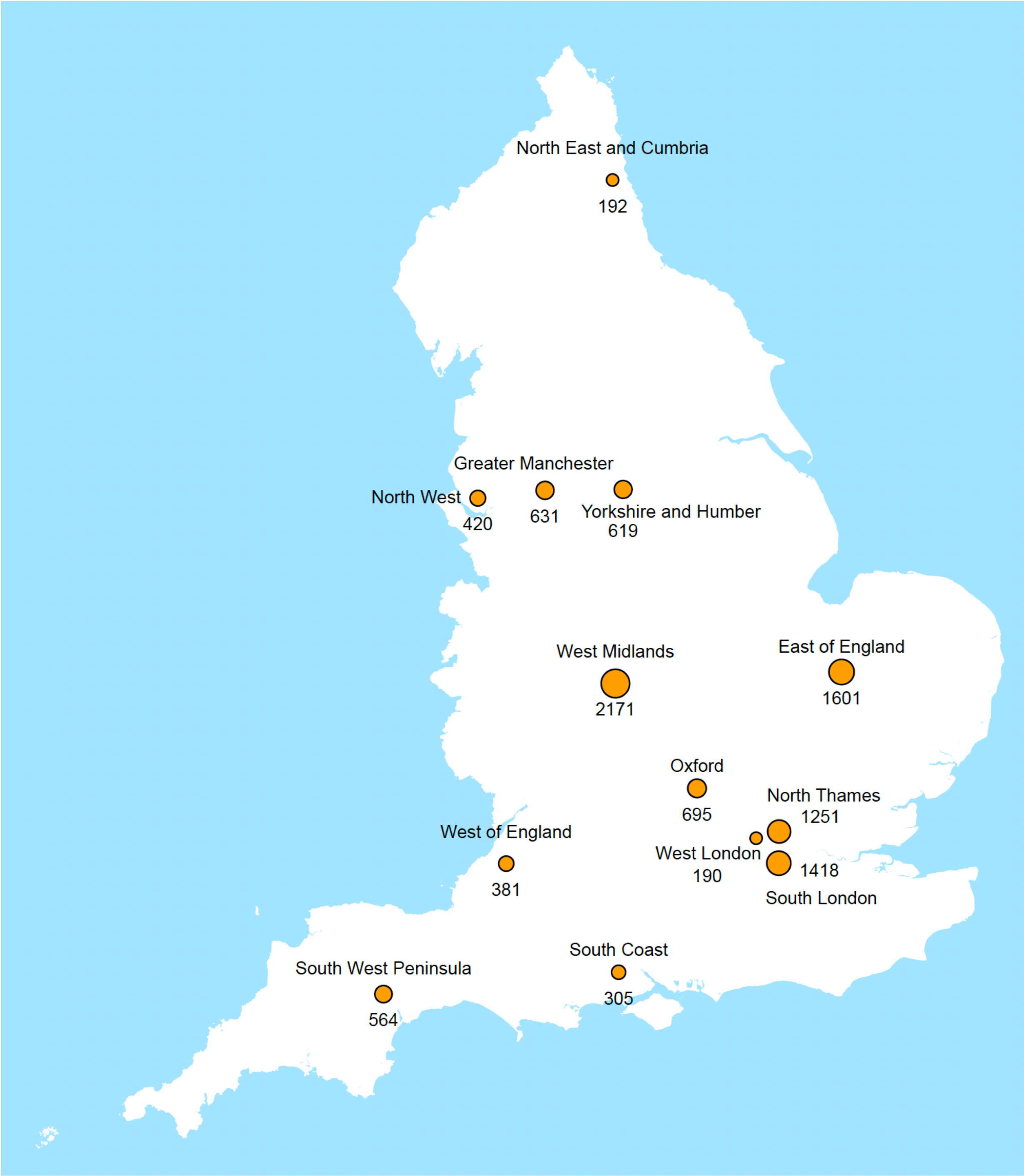

Supplementary Figure 9. Thirteen NHS Genomic Medicine Centres recruited patients diagnosed with cancer across England. The area of the circle is proportional to the number of patients recruited and the total number of participants recruited per GMC is indicated in brackets. 32 patients were also recruited from Northern Ireland.
