# Supplementary material for: Analysis of 10,478 cancer genomes identifies candidate driver genes and opportunities for precision oncology
Source: Nat Genet. 2024 Jun 18;56(9):1868–77. doi: 10.1038/s41588-024-01785-9 (PMC11387197; doi:10.1038/s41588-024-01785-9)
Supplement: Supplementary file 2 — Reporting Summary [file 41588_2024_1785_MOESM2_ESM.pdf]

nature portfolio

Corresponding author(s): Richard Houlston

Last updated by author(s): Mar 30, 2024

# Reporting Summary

Nature Portfolio wishes to improve the reproducibility of the work that we publish. This form provides structure for consistency and transparency in reporting. For further information on Nature Portfolio policies, see our Editorial Policies and the Editorial Policy Checklist.

## Statistics

For all statistical analyses, confirm that the following items are present in the figure legend, table legend, main text, or Methods section.

| n/a | Confirmed | |
|---|---|---|
| ☐ | ☒ | The exact sample size (*n*) for each experimental group/condition, given as a discrete number and unit of measurement |
| ☐ | ☒ | A statement on whether measurements were taken from distinct samples or whether the same sample was measured repeatedly |
| ☐ | ☒ | The statistical test(s) used AND whether they are one- or two-sided<br>*Only common tests should be described solely by name; describe more complex techniques in the Methods section.* |
| ☐ | ☒ | A description of all covariates tested |
| ☐ | ☒ | A description of any assumptions or corrections, such as tests of normality and adjustment for multiple comparisons |
| ☐ | ☒ | A full description of the statistical parameters including central tendency (e.g. means) or other basic estimates (e.g. regression coefficient) AND variation (e.g. standard deviation) or associated estimates of uncertainty (e.g. confidence intervals) |
| ☐ | ☒ | For null hypothesis testing, the test statistic (e.g. *F*, *t*, *r*) with confidence intervals, effect sizes, degrees of freedom and *P* value noted<br>*Give P values as exact values whenever suitable.* |
| ☒ | ☐ | For Bayesian analysis, information on the choice of priors and Markov chain Monte Carlo settings |
| ☒ | ☐ | For hierarchical and complex designs, identification of the appropriate level for tests and full reporting of outcomes |
| ☐ | ☒ | Estimates of effect sizes (e.g. Cohen's *d*, Pearson's *r*), indicating how they were calculated |

*Our web collection on statistics for biologists contains articles on many of the points above.*

## Software and code

Policy information about availability of computer code

**Data collection**

Samples were collected and processed by Genomics England. The code used for curation of samples is available inside the Genomics England Research Environment under /re_gecip/shared_allGeCIPs/pancancer_signatures/code/processClinicalData.

**Data analysis**

Details and code for using the Intogen framework are available here (https://intogen.readthedocs.io/en/latest/index.html). The specific code to perform this analysis is available in the Genomics England research environment under /re_gecip/shared_allGeCIPs/pancancer_drivers/code/. The link to becoming a member of the Genomics England research network and obtaining access can be found here https://www.genomicsengland.co.uk/research/academic/join-gecip. The code to perform the canSAR chemogenomics analysis is available through Zenodo (https://zenodo.org/record/8329054).
Additional packages/softwware used:
VerifyBamID v1.1.3 = https://github.com/statgen/verifyBamID
Ccube v1 = https://github.com/keyuan/ccube
Isaac aligner v03.16.02.19 = https://github.com/Illumina/Isaac3
Strelka v2.4.7 = https://github.com/Illumina/strelka
bcftools v1.9 = https://samtools.github.io/bcftools/bcftools.html
alleleCount-FixVAF v4.1.0 = https://github.com/danchubb/alleleCount-FixVAF
VEP v101 = https://github.com/Ensembl/ensembl-vep
CADD v1.6 = https://github.com/kircherlab/CADD-scripts/
OncoKb v3.11 = https://www.oncokb.org/api-access
trackViewer v1.38.2 = https://github.com/jianhong/trackViewer
mSINGS = https://bitbucket.org/uwlabmed/msings/src/master/
HRDetect = https://github.com/eyzhao/hrdetect-pipeline

Battenberg v2.2.8 =https://github.com/Wedge-lab/battenberg
Delly v0.7.9= https://github.com/dellytools/delly
Lumpy v0.2.13 = https://github.com/arq5x/lumpy-sv/releases
Manta v1.5.0 = https://github.com/Illumina/manta
GATK v.4.4.0 = https://github.com/broadinstitute/gatk
BEDOPS v2.4.2 = https://github.com/bedops/bedops
bedtools v2.3.0 = https://bedtools.readthedocs.io/en/latest/index.html
MutationTimeR v0.99.2 = https://github.com/gerstung-lab/MutationTimeR

For manuscripts utilizing custom algorithms or software that are central to the research but not yet described in published literature, software must be made available to editors and reviewers. We strongly encourage code deposition in a community repository (e.g. GitHub). See the Nature Portfolio guidelines for submitting code & software for further information.

## Data

Policy information about availability of data

All manuscripts must include a data availability statement. This statement should provide the following information, where applicable:

- Accession codes, unique identifiers, or web links for publicly available datasets
- A description of any restrictions on data availability
- For clinical datasets or third party data, please ensure that the statement adheres to our policy

Summary statistics for each tumour group are provided in the supplementary tables where such data does not enable identification of participants. All sample-specific WGS data and processed files from the 100,000 Genomes Project can be accessed by joining the Pan Cancer Genomics England Clinical Interpretation Partnership (GeCIP) Domain once an individual's data access has been approved (https://www.genomicsengland.co.uk/research/pan-cancer). The link to becoming a member of the genomics england research network and having access can be found here https://www.genomicsengland.co.uk/research/academic/join-gecip. The process involves an online application, verification by the applicant's institution, completion of a short information governance training course, and verification of approval by Genomics England. Please see https://www.genomicsengland.co.uk/research/academic for more information. The Genomics England data access agreement can be obtained from https://figshare.com/articles/dataset/GenomicEnglandProtocol_pdf/4530893/7. All analysis of Genomics England data must take place within the Genomics England Research Environment (https://www.genomicsengland.co.uk/understanding-genomics/data). The 100,000 Genomes Project publication policies can be obtained from https://www. genomicsengland.co.uk/about-gecip/publications. Samples and results used in this study are provided in Genomics England under /re_gecip/shared_allGeCIPs/pancancer_drivers/results/. A MAF-like file detailing coding mutations across all 100kGP tumours analysed is available at /re_gecip/shared_allGeCIPs/pancancer_drivers/results/. The COSMIC and OncoKB clinical actionability data are available from https://cancer.sanger.ac.uk/actionability and https://www.oncokb.org/actionableGenes#sections=Tx, respectively. The canSAR chemogenomics data are available from https://cansar.ai/. The NHS Genomic Test Directory for Cancer is available from https://www.england.nhs.uk/publication/national-genomic-test-directories/. List of drivers from prior studies obtained from COSMIC (https://cancer.sanger.ac.uk/cmc/home), Intogen (https://www.intogen.org/search) and and the The Cancer Genome Atlas (TCGA) Program pan-cancer analysis reported by Bailey et al. Somatic mutations were annotated to the cached version of GRCh38 in VEP v101.

## Research involving human participants, their data, or biological material

Policy information about studies with human participants or human data. See also policy information about sex, gender (identity/presentation), and sexual orientation and race, ethnicity and racism.

| | |
|---|---|
| Reporting on sex and gender | Sex was used as reported by NHSD, PHE/NCRAS and the GMCs where this matched the inferred sex from genomic sequencing. Where they do not match the sample was excluded. |
| Reporting on race, ethnicity, or other socially relevant groupings | Reported race, ethnicity, or other socially relevant groupings were not used in this study. |
| Population characteristics | Information relating to the cohort in this analysis are provided in supplementary table 3. The collection and processing of treatment information is described in detail in the methods. |
| Recruitment | Clinical and demographic data were obtained from NHS Digital (NHSD), Public Health England's National Cancer Registration and Analysis Service (PHE-NCRAS) and the Genomic Medicine Centres (GMCs) through the Genomics England Research Environment. |
| Ethics oversight | The 100,000 Genomes Project protocol was approved by the East of England and South Cambridge Research Ethics Committee on 20 February 2015, REC reference 14/EE/1112 |

Note that full information on the approval of the study protocol must also be provided in the manuscript.

# Field-specific reporting

Please select the one below that is the best fit for your research. If you are not sure, read the appropriate sections before making your selection.

☒ Life sciences ☐ Behavioural & social sciences ☐ Ecological, evolutionary & environmental sciences

For a reference copy of the document with all sections, see nature.com/documents/nr-reporting-summary-flat.pdf

# Life sciences study design

All studies must disclose on these points even when the disclosure is negative.

| | |
|---|---|
| Sample size | 10,478 samples were included in the full cohort. Exact sample sizes for tumour groups are provided in supplementary table 2. Sample size was chosen based on the availability of whole genome sequencing of tumour/normal pairs in the Genomics England research environment. |
| Data exclusions | A detailed description of the sample quality control is provided in the methods. Supplementary table 1 provides information on how many samples were excluded. Sequenced tumour samples were excluded if clinical data were missing or if unresolvable conflicts existed between the clinical data sources (GMCs, NHSD, PHE-NCRAS, histology reports). In total 2,251/14,129 (15.9%) of tumour samples were excluded based on availability and consistency of reported sex, tumour histology, tumour type, sampling date or if the participant was recorded as less than 18 years old at the time of sampling. 267/11878 (2.2%) of tumour samples with required clinical data available were excluded based on tumour sample purity and sequencing data quality. Duplicate tumour samples were also removed, to ensure that no individual was represented more than once in a tumour group. If multiple sequenced tumour samples from the same tumour group were available for an individual, we preferentially kept primary tumour samples with highest purity. Non-solid tumours were removed from this analysis. Based on these criteria, 10,478 tumour samples were suitable for analysis. |
| Replication | This study has an observational rather than an experimental study design, and only one sample was sequenced from each participant, in the great majority of cases. We replicate many of the findings from previously published studies of somatic cancer driver genes. |
| Randomization | This study has an observational rather than an experimental study design hence randomisation of study participants is not relevant. |
| Blinding | This study used real-world observation data collected from NHS trusts. The investigators did not have control over sample selection, collection and processing and as such blinding is not relevant to this study. |

# Reporting for specific materials, systems and methods

We require information from authors about some types of materials, experimental systems and methods used in many studies. Here, indicate whether each material, system or method listed is relevant to your study. If you are not sure if a list item applies to your research, read the appropriate section before selecting a response.

## Materials & experimental systems

| n/a | Involved in the study |
|---|---|
| ☒ | ☐ Antibodies |
| ☒ | ☐ Eukaryotic cell lines |
| ☒ | ☐ Palaeontology and archaeology |
| ☒ | ☐ Animals and other organisms |
| ☒ | ☐ Clinical data |
| ☒ | ☐ Dual use research of concern |
| ☒ | ☐ Plants |

## Methods

| n/a | Involved in the study |
|---|---|
| ☒ | ☐ ChIP-seq |
| ☒ | ☐ Flow cytometry |
| ☒ | ☐ MRI-based neuroimaging |

# Plants

| | |
|---|---|
| Seed stocks | *Report on the source of all seed stocks or other plant material used. If applicable, state the seed stock centre and catalogue number. If plant specimens were collected from the field, describe the collection location, date and sampling procedures.* |
| Novel plant genotypes | *Describe the methods by which all novel plant genotypes were produced. This includes those generated by transgenic approaches, gene editing, chemical/radiation-based mutagenesis and hybridization. For transgenic lines, describe the transformation method, the number of independent lines analyzed and the generation upon which experiments were performed. For gene-edited lines, describe the editor used, the endogenous sequence targeted for editing, the targeting guide RNA sequence (if applicable) and how the editor was applied.* |
| Authentication | *Describe any authentication procedures for each seed stock used or novel genotype generated. Describe any experiments used to assess the effect of a mutation and, where applicable, how potential secondary effects (e.g. second site T-DNA insertions, mosiacism, off-target gene editing) were examined.* |
